# Formal derivation of the Laughlin function and its generalization for other topological phases of FQHE

**DOI:** 10.1038/s41598-021-04672-z

**Published:** 2022-01-12

**Authors:** Janusz E. Jacak

**Affiliations:** Wrocław University of Science and Technology, Wyb. Wyspiańskiego 27, 50-370 Wrocław, Poland

**Keywords:** Topological matter, Quantum Hall

## Abstract

Using the braid symmetry we demonstrate the derivation of the Laughlin function for the main hierarchy 1/*q* of FQHE in the lowest Landau level of two-dimensional electron system with a mathematical rigour. This proves that the derivation of Laughlin function unavoidably requires some topological elements and cannot be completed within a local quantum mechanics, i.e., without global topological constraints imposed. The method shows the way for the generalization of this function onto other fractions from the general quantum Hall hierarchy. A generalization of the Laughlin function is here formulated.

## Introduction

Correlations in multi-electron systems are induced by the interaction of electrons which can drive various types of collective system organizations. In conventional scheme of phase transitions the correlated multiparticle state is assigned by local order parameter linked to a mass operator in some channel of coherent scattering of electrons and associated with a spontaneous breaking of some symmetry. In a superconductor, the U(1) gauge symmetry is broken via Cooper pairing. The related gap in single-particle spectrum plays the role of an order parameter and occurs due to binary attraction of electrons on the Fermi surface by virtue of exchange of phonons^1,2^. Breaking of spin rotation symmetry results in magnetic ordering in spin interacting systems in Heisenberg or Ising channels with local magnetization as the order parameter^3^. In ordered phase the Goldstone-type gap-less collective excitation usually occurs which restores the lost symmetry^4^. Nevertheless, in 2D systems these Goldstone modes disrupt the long range correlations at nonzero temperatures according to Mermin-Wagner theorem^5,6^, which dismisses a conventional phase transition in planar systems. Instead, a topological type ordering can occur in 2D multi-electron systems with most famous Kosterlitz-Thouless scheme for a phase transition^7^. Topological correlations are neither assigned by a local order parameter nor assisted with a symmetry breaking. To this class of topological phases belong also correlated states of fractional quantum Hall effect (FQHE) in 2D multi-electron systems exposed to quantizing perpendicular magnetic field^8^. FQHE has been experimentally discovered in 1982^9^ by Tsui, Stormer and Gossard in GaAs 2DES and the multiparticle wave function for the corresponding correlated state has been proposed by Laughlin in 1983^10^. Both these achievements have been distinguished with the Nobel prize in 1998.

The Laughlin function has been, however, proposed without a derivation^10,11^ in order to distinguish in a phenomenological manner the state of interacting 2D electrons at fractional filling of the lowest Landau level (LLL) from the gaseous system described by the Slater function constructed of single particle Landau states for the case of the completely filled LLL without interaction. This proposition resolved itself to the substitution of the Vandermonde polynomial, $\prod _{i,j;i>j}^N(z_i-z_j)$, in the Slater function for completely filled LLL of noninteracting 2D electrons by the Jastrow polynomial, $\prod _{i,j;i>j}^N(z_i-z_j)^q$, where *q* is an odd integer and $z_i$ is the *i*-th electron position on the plane expressed as the complex number. Such a function is not any more the Slater determinant but it occurred to be an almost perfect approximation of the ground state for interacting electrons at fractional filling $\nu =\frac{1}{q}$ of the LLL, which has been verified with accuracy ca. 99% by exact diagonalization of the Coulomb interaction in toy planar system with 3 electrons^10,11^, and next also verified for higher number of electrons, up. to ca 20. Though some additional motivation by thermodynamic analogy have been presented^10,12^, none derivation of the Laughlin function has been provided. The lack of the derivation of the Laughlin function causes the basic problem with understanding of the physics related to the described by this function FQHE states, interpreted also only phenomenologically, e.g., using the model of composite fermions, hypothetical particles composed of electrons and attached to them quanta of flux of auxiliary but fictitious magnetic field^13^. Other various trials to derive or interpret the Laughlin function have been also undertaken, including conformal field theory approach using so-called Chern-Simons gauge field^14–16^, vortex approach by Read^17^, multi-component development of the Laughlin function by Halperin^18^, pseudopotential approximation by Haldane^19^ or algebraic approach using Jack polynomials^20^, chiral boson approach and others. In particular, it has been proved that the Laughlin function with the exponent *q* in the Jastrow polynomial is the exact ground state if the so-called Haldane pseudopotentials (the matrix elements of Coulomb interaction of an electron pair with relative angular momentum *m*) are neglected for $m>q-2$^19,21^. All these approaches did not, however, supply a complete derivation of the Laughlin function.

In the present paper we formulate the derivation of the Laughlin function with the mathematical rigour from the first rules utilizing the braid symmetry of multi-electron interacting 2D systems in the presence of magnetic field. We base on our formerly developed braid group approch to FQHE^8,22^, but the detailed derivation of the Laughlin function is original. The method occurs to be sufficiently meaningful for the generalization of the derivation onto other filling fractions at which FQHE effect has been observed apart from simple fractions $\frac{1}{q}$, and for which the analytical exact wave-function-forms of the multiparticle ground states have not been known. We propose a general form of the multi-electron wave function for various homotopy phases of interacting 2D electrons corresponding to an arbitrary filling fraction from the general hierarchy of FQHE in the LLL, again with all the details presented for the first time in general case and for explicit examples related to the filling fractions both of composite fermion type and of so-called enigmatic FQHE states in the LLL. The energies of these exemplary fractional states have been assessed for varying number of electrons.

## Landau levels at rotational symmetry gauge

For the symmetric gauge of magnetic field ${\mathbf {B}}=(0,0,B)$ perpendicular to a plane (*x*, *y*),

1 $$\begin{aligned} {\mathbf {A}}=\frac{B}{2}(-y,x,0), \end{aligned}$$

the rotational invariance is preserved (infinite planar multi-electron system exposed to perpendicular magnetic field supports both translation and cylindrical symmetry, and we choose the gauge consistent with circular shape of the finite sample with the surface $S=\pi R^2$). Let us consider first a single 2D electron problem^19,23^. One can introduce the mutually conjugated operators (of annihilation and creation type),

2 $$\begin{aligned} a=-i \sqrt{\frac{\hbar c}{2eB}}\left( 2{\bar{\partial }} +\frac{eB}{2\hbar c} z\right) \end{aligned}$$

and

3 $$\begin{aligned} a^+=-i\sqrt{\frac{hc}{2eB}}\left( 2\partial -\frac{eB}{2\hbar c}{\bar{z}} \right) , \end{aligned}$$

where $\partial =\frac{\partial }{\partial z}=\frac{1}{2}(\nabla _x-i\nabla _y)$, ${\bar{\partial }}=\frac{\partial }{\partial {\bar{z}}}=\frac{1}{2}(\nabla _x+i\nabla _y)$, $z=x+iy$, ${\bar{z}}=x-iy$ (*z* denotes the electron position on the plane represented as the complex number). Note that $\partial z={\bar{\partial }}{\bar{z}}=1$, $\partial {\bar{z}}={\bar{\partial }}z =0$ (thus *z* and ${\bar{z}}$ are independent variables in partial derivatives). One can check that $[a,a^+]=1$. Landau Hamiltonian of a single 2D electron in magnetic field has the form,

4 $$\begin{aligned} H=\hbar \omega \left( a^+a+\frac{1}{2}\right) ,\;\;\omega =\frac{eB}{mc}, \end{aligned}$$

and its ground state is the solution of the equation,

5 $$\begin{aligned} a\left| 0\right\rangle =-i\sqrt{\frac{\hbar c}{2eB}}\left( 2{\bar{\partial }} +\frac{eB}{2\hbar c}z\right) \psi (z,{\bar{z}})=0. \end{aligned}$$

The solution of the above equation attains the form,

6 $$\begin{aligned} \psi (z,{\bar{z}})=f(z)e^{-eBz {\bar{z}}/4\hbar c}=f(z)e^{-z{\bar{z}}/4l_B^2}, \end{aligned}$$

where *f*(*z*) is an arbitrary analytic function, $l_B=\sqrt{\frac{\hbar c}{eB}}$ is the so-called magnetic length—the length-scale at magnetic field presence. The freedom in choice of *f*(*z*) function displays the degeneracy of LLs. One can choose *f*(*z*) as independent monomials $z^n$ (*n* non-negative integer)—the basis of analytic Taylor decomposition (though other bases are also possible). Then,

7 $$\begin{aligned} \psi _n(z,{\bar{z}})={\mathscr {A}}_nz^n e^{-z{\bar{z}}/4l_B^2}, \end{aligned}$$

with the normalization condition ($z{\bar{z}}=r^2$, i.e., the square of cylindrical radius),

8 $$\begin{aligned} \begin{array}{l} {\mathscr {A}}_n^2\int dr 2\pi rr^{2n}e^{-eBr^2/2\hbar c}={\mathscr {A}}_n^2\pi \left( \frac{2\hbar c}{eB}\right) ^{n+1} \Gamma (n+1)\\ \quad ={\mathscr {A}}_n^2\pi \left( \frac{2\hbar c}{eB}\right) ^{n+1}n!=1,\\ \end{array} \end{aligned}$$

which gives ${\mathscr {A}}_n=\left( n! \pi \left( \frac{2\hbar c}{eB}\right) ^{n+1}\right) ^{-1/2}$. Note that, $\left\langle r^2\right\rangle= {\mathscr {A}}_n^2 \int du u^{n+1}e^{-eBu /2\hbar c}= (n+1)\frac{2\hbar c}{eB}$, $u=r^2$, the average radius squared is further away from the origin for larger *n*. From the boundary condition $r_{max}^2=(2n+1)\frac{\hbar c}{eB}<R^2$ for the circular sample with radius *R*, one gets $n\le \frac{eB\pi R^2}{hc}$, i.e., the total magnetic flux $B\pi R^2$ divided by magnetic field flux quantum $\frac{hc}{e}$. The maximal value of radius, $r_{max}$ is determined from the condition $\frac{d}{dr} 2\pi r|\psi _n|^2=0$, which gives $r_{max}^2=(2n+1)\frac{\hbar c}{eB}$.

Excited states can be found by acting the creation operator $a^+$ on the ground state, e.g., the first exited states (indexed by *n*) are,

9 $$\begin{aligned} a^+\psi _n=-i \sqrt{\frac{\hbar c}{2eB}}\left( 2n-\frac{{\bar{z}} z}{l_B^2}\right) {\mathscr {A}}_nz^{n-1}e^{-{\bar{z}}z/4l_B^2}, \end{aligned}$$

with the same energy $E_1=\frac{3}{2}\hbar \omega$ for all *n* (whereas the ground state energy of Hamiltonian (4) was $E_0=\frac{1}{2}\hbar \omega$).

## The Laughlin function

If one considers *N* electrons on a plane neglecting their interaction (the gas) and exposed to the perpendicular magnetic field, then the state for completely filled LLL must be described by the Slater function of degenerate ground states of a single electron, which differ between them only by factor $z^n$ (and by unimportant normalization constant) as demonstrated in Eq. (7). Thus the Slater function attains the shape of the Vandermonde determinant,

10 $$\begin{aligned} {\mathscr {B}}\; \left| \begin{array}{lllll} 1&{}z_1&{}z_1^2&{}\ldots &{}z_1^{N-1}\\ 1&{}z_2&{}z_2^2&{}\ldots &{}z_2^{N-1}\\ \ldots &{}\ldots &{}\ldots &{}\ldots &{}\ldots \\ 1&{}z_N&{}z_N^2&{}\ldots &{}z_N^{N-1}\\ \end{array} \right| e^{- \frac{eB}{4\hbar c}\sum _i^N\bar{z_i}z_i}={\mathscr {B}} \prod _{i,j;i<j}^N(z_i-z_j)e^{-\sum _i^Nr_i^2/4l_B^2}, \end{aligned}$$

where $z_i$ is the complex coordinate of *i*-th electron on the plane, $l_B=\sqrt{\frac{\hbar c}{e B}}$ is the magnetic length and the number of electrons $N=N_0=\frac{BSe }{hc}$, $S=\pi R^2$ is the surface of a circular sample, *B* is the external perpendicular magnetic field, $N_0$ is the degeneracy of LLs, ${\mathscr {B}}$ is the normalization constant.

Tha Laughlin *ansatz* consists in the substitution of the the Vanderomode polynomial in (10) by the Jastrow polynomial, i.e., Laughlin substituted the factor $(z_i-z_j)$ by the factor $(z_i-z_j)^q$, *q*—odd integer. The Laughlin function has thus the form,

11 $$\begin{aligned} {\mathscr {C}} \prod _{i,j;i<j}^N(z_i-z_j)^qe^{-\sum _i^Nr_i^2/4l_B^2}, \end{aligned}$$

where ${\mathscr {C}}$ is the normalization constant, and Laughlin argued that $N= \frac{N_0}{q}$, i.e., the function (11) corresponds to the filling fraction of the LLL, $\nu =\frac{N}{N_0}=\frac{1}{q}$, in the case of Coulomb *interacting* electrons (at the magnetic field *q* times larger than that for the function (10) at the same *N* and *S*, as the degeneracy of LLs is proportional to magnetic field *B*).

This function appeared to be correct and it has been demonstrated that it agrees with exact diagonalization of Coulomb interaction in small finite models of few electrons at $\nu =\frac{1}{q}$ with accuracy ca. 99% (a little discrepancy is connected rather with inaccuracy of the exact diagonalization along the Lanczos method in this case). The derivation of Eq. (11) had never been done.

We will argue that the formal derivation of the Laughlin function needs topological methods and thus was not possible upon the local quantum mechanics.

Instead of conducting a derivation Laughlin motivated that the square modulus of the function (11) has the shape of the thermodynamic Boltzmann distribution function,

12 $$\begin{aligned} {\mathscr {C}}^2 exp\left[ -\frac{1}{q} \left( -2q^2 \sum _{i<j}^N\operatorname{ln}|z_i-z_j|+\frac{q}{2}\sum _i^N\frac{|z_i|^2}{l_B^2}\right) \right] . \end{aligned}$$

Formally, this is the classical Boltzmann distribution function for the two-dimensional homogeneous plasma of particles with charge *q* at the temperature $T=\frac{q}{k_B}$ ($k_B$ is Boltzmann constant)^12^. The term with logarithm corresponds to Coulomb interaction in 2D of *i*-th and *j*-th particles with charges *q*, $\sim -q^2\operatorname{ln}|z_i-z_j|$, whereas the second term in the exponent mimics the interaction of particles with oppositely charged jellium with the charge density $\frac{1}{2\pi l_B^2}$. The equilibrium for such a plasma system is attained when the system is electrically balanced, i.e., when $\frac{1}{2\pi l_B^2}=q\frac{N}{S}$ (where *S* is the sample surface), which gives the filling fraction $\nu =\frac{N}{N_0}=\frac{N}{(BSe/hc)}=\frac{1}{q}$. Thus, the maximal value of the Laughlin function is also attained at filling fraction $\nu =\frac{1}{q}$.

As each *N*-electron wave function (including interaction) can be decomposed on the basis of Slater functions of noninteracting *N*-particle states from the LLL (if mixing with next LLs is precluded), then such a function must be of the general form,

13 $$\begin{aligned} g(z_1,\dots , z_N)e^{-\sum _i^N|z_i|^2/4l_B^2}, \end{aligned}$$

where $g(z_1,\dots ,z_N)$ is an polynomial (which is a consequence of Eq. (7) and next of Eq. (10)). The function $g(z_1,\dots ,z_N)$ must be a holomorphic function (analytic function on the whole domain) due to form of the single particle LLL states, which are given by Eq. (6) with analytic factor *f*(*z*) (without the loss of generality, conventionally assumed as $z^n$), thus, in general, possible to be decomposed into power series in the whole domain. The envelope function $e^{-\sum _i^N|z_i|^2/4l_B^2}$ is common for an arbitrary *N*-electron function in the LLL ($N\le N_0$). The total angular momentum of electrons, which is given by the degree of the polynomial $g(z_1,\dots ,z_N)$, is a good quantum number for Coulomb interacting particles, thus the eigen-function of the total angular momentum must be a homogeneous polynomial. This is, however, too loose to restrict the form of polynomial $g(z_1,\dots ,z_N)$. Laughlin, assuming a perfect antisymmetry of *g* (addressed to the Pauli principle), guessed this polynomial in the form of Jastrow function,

14 $$\begin{aligned} g(z_1,\dots , z_N)=\prod _{i,j;i>j}^N(z_i-z_j)^q,\;\; q \text{ - odd positive integer}. \end{aligned}$$

However, the derivation of (14) was never provided.

Below we will demonstrate that a proof of the choice in form of (14) unavoidably requires some topological arguments, which make the derivation complete and mathematically rigorous. Moreover, the topological approach can be generalized onto other filing fractions of FQHE hierarchy (apart from only $\frac{1}{q}$).

## Derivation of the Laughlin function

It has been proved^24–26^ that the multiparticle wave function $\Psi ({\mathbf {r}}_1, \dots , {\mathbf {r}}_N)$ must transform according to scalar unitary representation of the braid if coordinates of this function transpose their positions in the manner prescribed by this braid (cf. “Appendix 1”). Braid groups are collections of nonhomotopic classes of loops in the multiparticle configuration space (the closed *N*-thread trajectories, which cannot be transformed one onto another one by any continuous deformation without cutting)^24,27–30^. The configuration space of *N* identical indistinguishable particles is defined as follows,

15 $$\begin{aligned} F_N=(M^N-\Delta )/S_N, \end{aligned}$$

where *M* is the physical space (mathematically a manifold) on which all particles are located (it can be 3D space ${\mathbb {R}}^3$, or 2D space ${\mathbb {R}}^2$ or other manifolds like the surface of a sphere or of a torus), $M^N=M\times M\times M \times \dots \times M$ is *N*-fold product of the manifold *M*, $\Delta$ is the diagonal subset of $M^N$ in which the coordinates of at least two particles coincide, and $\Delta$ set is subtracted in order to ensure the particle number conservation. The division by the permutation group $S_N$ introduces the indistinguishability of particles, i.e., the points in the configuration space $F_N$, which differ only by a permutation of particle positions, coincide after the division by $S_N$. The space $F_N$ is always multiply connected (for $N\ge 3$)^27,31^ and its first homotopy group $\pi _1(F_N)$ called as the full braid group is nontrivial^27,29^.

Scalar unitary representations of the braid group assign quantum statistics of particles in the space $F_N$^27,28,30^ (cf. “Appendix 1” for explanation). Various representations define different quantum particles corresponding to the same classical ones. In this way one can rationalize bosons and fermions for *M* with dimension $>2$ and anyons for ${\text {dim}} M=2$^24,26,32^. The difference between 2D systems and higher dimensional ones originates from the fact that for ${\text {dim}} M>2$ the braid group is always the finite group $S_N$ (the permutation group of *N* elements) with only two unitary representations, $e^{i0}$ (bosons) and $e^{i\pi }$ (fermions), whereas for $M=R^2$ the full braid group is the infinite Artin group^29,33^ with scalar unitary representations $e^{i\alpha }, \;\alpha \in [0,2\pi )$ (anyons).

**Figure 1:**
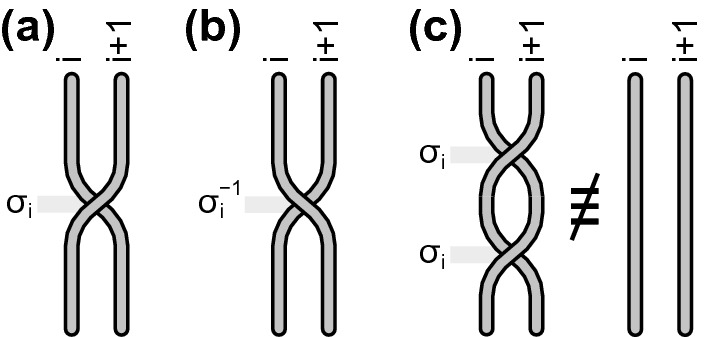
(**a**) Conventional geometric presentation for the generator $\sigma _i$ of the Artin group^29,33^—this generator describes the transposition of a particle *i*-th with $(i+1)$-th one on the plane $R^2$ when other particles remain on their positions. (**b**) Inverse braid $\sigma _i^{-1}$. (**c**) Square of generator $\sigma _i^2$, which for $M=R^2$ is not a neutral element of the group (though for $M=R^3$, $\sigma _i^2=e$ and this a reason of simplicity of the braid group in 3D in contrary to 2D case).

**Figure 2:**
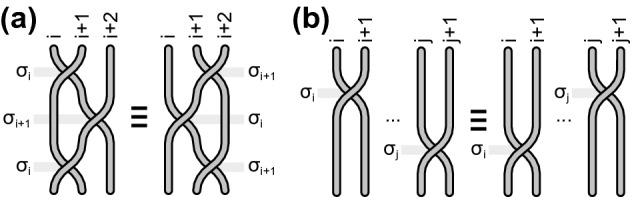
Formal conditions for generators, defining Artin group, i.e., the full braid group for $M=R^2$, (**a**) the graphical presentation of Eq. (16) in the convention as in Fig. 1 and (**b**) the graphical presentation of Eq. (17).

Nevertheless, in the case when $M=R^2$ and at the presence of magnetic field the situation changes considerably. The braid group structure becomes here much more complicated^8^. On 2D plane the positions of particles (${\mathbf {r}}_i$) can be represented as complex numbers $z_i$ and exchanges of particle positions in $F_N$ can be imagined as transpositions of points on the complex plane. Let us first consider such a picture for the Artin group, $\pi _1(F_N)=\pi _1((M^N-\Delta )/S_N), \;M=R^2$. This group is an infinite multi-cyclic group generated by $N-1$ generators, $\sigma _i$, $i=1,\dots , N-1$ describing exchanges of *i*-th particle with $(i+1)$-th one on the plane (at arbitrary, but fixed, numeration of particles being equivalent one to another due to particle indistinguishability). The braid corresponding to $\sigma _i$ is depicted in Fig. 1 using the conventional graphical presentation of braids^24,27,29^. The Artin group can be defined as the abstract group generated by elements $\sigma _i$, $i=1,\dots ,N-1$, which satisfy two conditions^29,33^,

16 $$\begin{aligned} \sigma _i\sigma _{i+1}\sigma _i= \sigma _{i+1}\sigma _{i}\sigma _{i+1},\; \quad {\mathrm{for}}\; 1\le i\le N-2,\;\;\;\;\; \end{aligned}$$

17 $$\begin{aligned} \sigma _i\sigma _j = \sigma _j\sigma _i,\; \quad \mathrm{for}\;1\le i,j\le N-1,\; |i-j|\ge 2, \end{aligned}$$

the graphical presentation of above conditions is shown in Fig. 2. For Artin group it follows from Eq. (16) that scalar unitary representations of $\sigma _i$ are independent of *i* (because by virtue of (16) $\sigma _{i}=\sigma _{i+1}\sigma _i\sigma _{i+1}\sigma _i^{-1}\sigma _{i+1}^{-1}$ and due to the commutation of scalar representations, $e^{i\alpha _i}=e^{i\alpha _{i+1}}=e^{i\alpha }$, where $e^{i\alpha _i}$ is the scalar unitary representation of $\sigma _i$, i.e., $\sigma _i\rightarrow e^{i\alpha _i}=e^{i\alpha }$). For $M=R^3$ (or higher dimensional space) the full braid group is the permutation group $S_N$, for which $\sigma _i^2=e$ (neutral element). Thus for $S_N$ scalar unitary representation must be only $\sigma _i\rightarrow \pm 1$.

In the case of electrons on the plane exposed to perpendicular magnetic field braids have, however, a specific cyclotron shape as no other trajectories exist at magnetic field presence. Moreover, the planar braids acquire a finite spatial metric because planar cyclotron orbits of electrons are of a finite size, so braids obligatory built of pieces of cyclotron orbits are also of the same finite size. This metric we mean here a spatial finite range of the braid trajectories (no axiomatic metric as in metric spaces) because these trajectories are halves of a cyclotron orbits of 2D electrons exposed to perpendicular magnetic field^34^, which on the plane are of a finite size. For 2D electrons their cyclotron orbits must lie on the plane and their surface area is defined by the magnetic field flux quantum divided by magnetic field—cf. “Appendix 2”. Moreover, the area of these cyclotron orbits is immune to deformations of orbits caused by electron interaction in multi-electron system, as proved by application of the Bohr–Sommerfeld rule^22^—this proof is summarized in “Appendix 2”. The limitation of the size of the cyclotron orbits of the 2D electrons causes the same confinement of the braid size as braids at magnetic presence must be built of fragments of cyclotron orbits (as no other trajectories are available at magnetic field presence). Note that in 3D cyclotron movement is not limited because of possible drift along the magnetic field direction and none metric can be imposed on 3D braids at magnetic field presence.

**Figure 3:**
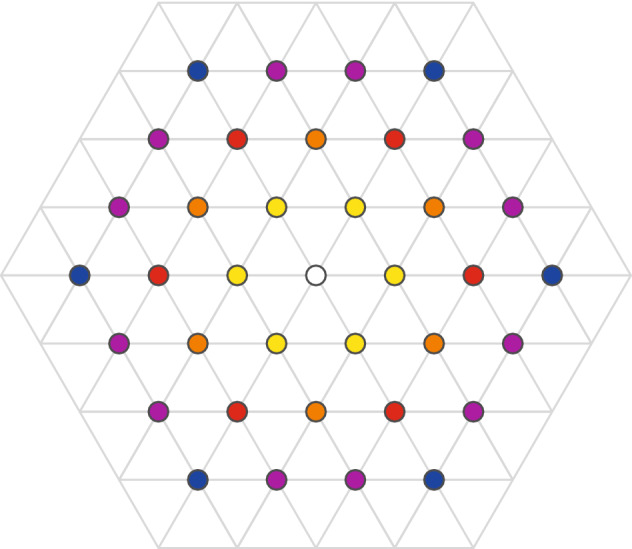
Classical 2D Wigner lattice of electrons with lowest energy of their interaction (at $T=0$ K when the classical kinetical energy is vanished). The lattice is hexagonal one with the elementary cell consisted of two triangles. The nearest neighbours and next-nearest neighbours up to 4-th rank are marked with different colours, cf. Ref.^8^.

**Figure 4:**
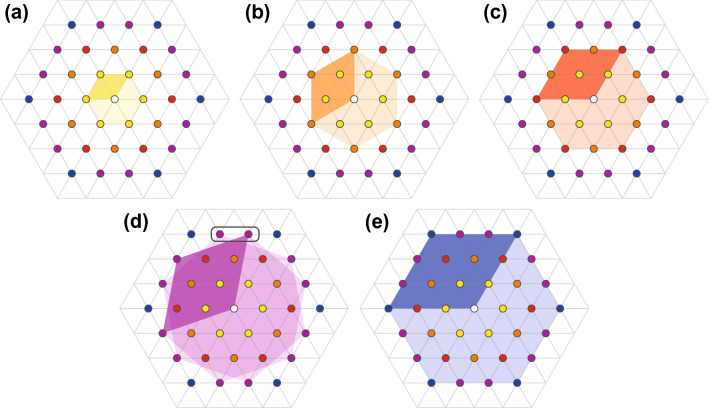
Bravais elementary cells in hexagonal sublattices of nearest (**a**) and next-nearest (**b**–**e**) neighbours in the classical Wigner lattice from Fig. 3 for 4 first ranks of neighbours (cf. Ref.^8^ for more detail).

In 2D gas of non-interacting particles the described above metric of braids does not impose any topological restriction because distances between gaseous particles can be arbitrary. However, if the interaction between 2D particles is switched on (like for electrons on the plane where they mutually interact via Coulomb forces) the situation is changing^8^. Repulsing electrons deposited on the positive uniform jellium (ensuring the balance of charges in total) create in 2D at $T=0$ K the classical Wigner hexagonal crystal—the triangle lattice of electron positions, which minimizes their repulsion energy (cf. Fig. 3, in which the hexagonal Wigner lattice is presented with in colour distinguished nearest and next-nearest neighbours up to 4-th rank). In Fig. 4 there are illustrated Bravais elementary cells for consecutive sublattices in classical Wigner lattice spanned by nearest and next-nearest neighbours up to 4-th rank. Sizes of consecutive Bravais cells define the surface per electron belonging to next-nearest neighbours electron subsets. As visible in Fig. 4 (and clarified in more detail in Ref.^8^), these subsets count $N/3,\;N/4,\;N/7,\;N/9$ electrons, which corresponds to the sizes of four first elementary cells of next-nearest neighbours of four first ranks, $S=ah,\;3ah, \;4ah, \;7ah,\;9ah$, where *a* is the side of the smallest triangle in the Wigner lattice and $h=\frac{\sqrt{3}}{2}a$ is the height in this triangle—cf. Figs. 3 and 4.

The presented above Wigner lattice is the classical distribution of electrons on the plane (when the kinetical energy is quenched at zero temperature) with minimal energy of Coulomb inter-particle interaction. In this case all braids from the braid group must be commensurate with the Wigner crystal, as the braids describe exchanges of electrons on the plane. This commensurability of braids with distribution of electrons on the plane is of central significance because braids describe exchanges of electrons (here on the plane) and in the case when the braid size is finite and precisely defined by the metric imposed by the 2D cyclotron orbit size, these braids must perfectly fit to electron position in the classical Wigner lattice. Otherwise the braids cannot be defined as being too short or too long in comparison to electron positions.

**Figure 5:**
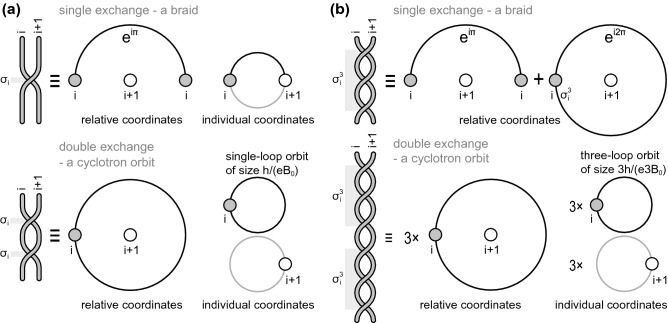
Schematic illustration that the elementary braids (generators) in the Artin group, $\sigma _i$, and in its cyclotron subgroups, $\sigma _i^q$ (in the figure for $q=3$), must be half-pieces of cyclotron orbits. The illustration is shown in individual coordinates of two particles in the pair on the plane and in relative coordinates, both for braid group generators—the elementary braids and for cyclotron orbits—the square of braids.

In particular the generators $\sigma _i$ can be implemented (i.e., defined) exclusively in the case when the cyclotron size of $\sigma _i$ (described above metric of this braid) perfectly coincides with the distance between nearest electrons in the Wigner lattice (at electron enumeration that $i+1$ denotes the closest neighbour of *i*-th electron in the lattice; such an enumeration is possible as it is evident for a selected electron in the Wigner lattice and all electrons are indistinguishable, thus it holds for all electrons). This cyclotron braid commensurability one can express in terms of the surface per particle in the Wigner lattice $\frac{S}{N}$ (*S* is the surface of the sample, *N* is the number of electrons in this sample, both kept fixed and constant) which must coincide with the surface of the cyclotron orbit. Such a definition of the commensurability is invariant with respect to interaction variation because the surface of cyclotron orbits is invariant to interaction—cf. “Appendix 2”. We avoid thus the problem of deformation of cyclotron planar orbits by the interaction of electrons. Considering the nearest neighbour electrons in the Wigner lattice we get thus the homotopy invariant (the cyclotron braids $\sigma _i$ fit perfectly to nearest neighbours in the Wigner lattice),

18 $$\begin{aligned} \frac{S}{N}=\frac{hc}{eB}, \end{aligned}$$

where $\frac{hc}{eB}$ is the size of the singleloop cyclotron orbit in the LLL (this is the magnetic flux quantum $\frac{hc}{e}$ divided by the magnetic field *B*)—cf.^8,35^. Note that the braid $\sigma _i$ is the half-piece of the cyclotron orbit as schematically illustrated in Fig. 5^34^. The homotopy in the name of this invariant emphasizes the fact that it has a topological nonlocal character related to the class of the homotopy of trajectories in the system controlled by the braid group – the first homotopy group of the multiparticle configuration space. Both sides of. Eq. (18) are invariant to interaction, the surface portion per single particle, $\frac{S}{N}$ as well as the planar cyclotron orbit surface area $\frac{hc}{eB}$ rigidly fixed by magnetic flux quantum $\frac{hc}{e}$.

The scalar unitary representation of $\sigma _i$ (the Artin group generators) has the form $e^{i\pi }$ for electrons (the same for all $i=1,\dots , N-1$), as electrons are fermions and from the general representation $e^{i\alpha }$ we must choose $\alpha =\pi$^27,29^. As the multiparticle wave function (13) must transform according to this representation when *i*-th particle transposes with $(i+1)$-th one, thus the function (13) must contain the factors,

19 $$\begin{aligned} z_i-z_{i+1}, \end{aligned}$$

where $i=1,\dots , N-1$ and $i+1$ denotes the nearest neighbour in the Wigner lattice with respect to *i*-th electron. This is clear, as the transposition of *i*-th particle with $(i+1)$-th one resolves itself to the rotation by $\pi$ of the complex number $z_i-z_{i+1}$. i.e., the transposition of these particles must give $e^{i\pi }(z_i-z_{i+1})$, which agrees with the unitary representation $e^{i\pi }$. This representation causes the shape of the factor (19) which must contribute to the multiparticle wave function. The situation here is trivial as the representation $e^{i\pi }$ of the generator $\sigma _i$ of the Artin group coincides in this special case with the fermionic representation of the permutation group $S_N$. Hence, in this case the factor (19) displays ordinary antisymmetry when variables of the multiparticle wave-function are exchanging. For 2D electron wavefunctions the exchange of their arguments (classical positions of electrons) is, however, not a simple permutation in general, which manifest itself by other scalar unitary representations corresponding to more specific braid groups. Other form of this unitary representation will force other form of the factor contributing to the multiparticle wave function than that given by (19), as will be illustrated below—cf. Eq. (25).

**Figure 6:**
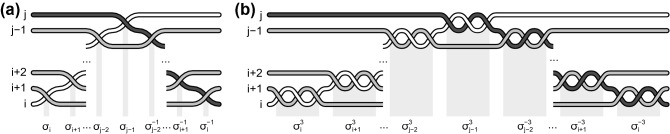
(**a**) The braid given by Eq. (20) defining the transposition of arbitrary particles *i*-th and *j*-th ones upon the structure of Artin group. (**b**) The braid given by Eq. (26) defining the transposition of particles *i*-th and *j*-th ones upon the cyclotron braid subgroup with $q=3$.

Now let us consider in this simplest case of the factor (19) its generalization for two arbitrarily enumerated particles, let say *i*-th and *j*-th ones. Then the braid realizing exchange of arbitrary *i*-th and *j*-th particles has the form,

20 $$\begin{aligned} \sigma _i\sigma _{i+1}\sigma _{i+2}\dots \sigma _{j-1}\sigma _{j-2}^{-1}\dots \sigma _{i+2}^{-1}\sigma _{i+1}^{-1} \sigma _{i}^{-1} \end{aligned}$$

and this braid is visualized in Fig. 6a. Scalar unitary representation of this long braid is obviously also $e^{i\pi }$ (as for each generator it is $e^{i\pi }$ and scalar representation is Abelian). Thus the pair of particle coordinates $z_i$ and $z_j$ must enter the polynomial *g* in Eq. (13) as the factor

21 $$\begin{aligned} z_i-z_j, \end{aligned}$$

due to the same arguments as for (19). Hence, the polynomial *g* in (13) must be of the form,

22 $$\begin{aligned} \prod _{i,j;i>j}^N(z_i-z_j), \end{aligned}$$

and this shape is the uniquely determined homogeneous polynomial (it cannot contain any other multiplicative factor of $z_i$ enhancing its rank).

This completes the derivation of the Laughlin function for *interacting* electrons at the field when singleloop braids perfectly fit to nearest neighbours in the Wigner lattice. In this case the homotopy invariant (18) can be equivalently rewritten as,

23 $$\begin{aligned} \frac{Nhc}{BSe}=1\;\rightarrow \; \nu =\frac{N}{N_0}=1, \end{aligned}$$

which shows that the field protected by the homotopy invariant (18) corresponds to the completely filled LLL. It must be emphasized that the Laughlin function with $q=1$ as derived it above defines the ground state of strongly correlated state of interacting electrons on the plane (this is the state for integer quantum Hall effect (IQHE)), though the shape of this multiparticle wave function coincides with the Slater function of *N* noninteracting fermions (10). The same wave function corresponds here to two different systems, with and without electron interaction. In the first case this wave function is the ground state of interacting electrons, thus strongly correlated, whereas in the second case the same wave function describes the completely filled LLL of the gas without any correlations induced by interaction (which is absent in a gas). Thus we see, that the correlations protected by the homotopy invariant (18) is not built in the wave function in this exceptional case of $\nu =1$. Or, in other words, the correlations induced by the Coulomb interaction at $\nu =1$ are topologically indistinguishable from Pauli correlations of noninteracting electrons at the complete filling of the LLL.

However, if the magnetic field changes, then the multiparticle wave function of interacting electrons also changes. Let us consider *q* times stronger field *B*. For such a field the filling fraction of the LLL diminishes *q* times as the LL degeneracy is proportional to the magnetic field.

But at so strong magnetic field the singleloop ordinary cyclotron orbits, $\frac{hc}{eB}$, are too small to reach even closest electrons in the Wigner lattice. It has been proved by application of the Bohr–Sommerfeld rule^22,35^ (cf. “Appendix 2” for short summary of this proof) that in the case when the braids $\sigma _i$ cannot be implemented as too short, the role of the elementary braids (transpositions of nearest neighbours in the Wigner lattice) take the braids $\sigma _i^q$ (*q*—odd positive integer). Such braids also describe the exchange of neighbouring particles but with additional $\frac{q-1}{2}$ loops. In the case when $\sigma _i$ braids cannot be implemented, the braids $\sigma _i^q$ have larger metric equal to $\frac{qhc}{eB}$^8,35^, cf. “Appendix 2”. Such larger braids are thus protected by the homotopy invariant,

24 $$\begin{aligned} \frac{S}{N}=\frac{qhc}{eB}. \end{aligned}$$

By comparison of (18) and (24) we see that the latter is fulfilled by the field *B* greater *q* times than that required for the former condition (18). The correlation defined by the invariant (24) defines the state for FQHE at $\nu =\frac{1}{q}$. Note that the Bohr–Sommerfeld rule applied to identify the effective magnetic flux quanta in many-particle correlated systems is independent of particle interaction and it holds for arbitrarily strongly or weakly interacting multiparticle systems (as the quasiclassical approach is not of perturbative type with respect to interaction). Hence, the values of effective quanta of magnetic flux are also interaction independent (invariant) for all various homotopy classes, although the selection of possible trajectories in (*x*, *y*) space is conditioned by the Coulomb repulsion of 2D charged particles (which allows the definition of the classical Wigner crystal needed to introduce the cyclotron commensurability). In the gas system of noninteracting particles their mutual positions are arbitrary, which dismisses any cyclotron commensurability and multiloop cyclotron orbits.

The new braid generators $\sigma _i^q$ for $i=1,\dots , N-1$, i.e., the simplest now exchanges of nearest-neighbours in the Wigner lattice, generate the subgroup of the original full braid group. This subgroup we call as the cyclotron subgroup^22^. Scalar unitary representations of the cyclotron subgroup are projective representations of the full braid group confined to the subgroup. These representations of cyclotron subgroups have the form $e^{iq\alpha }$, and for original fermionic electrons attain the form $e^{iq\pi }$. Thus, the multiparticle wave function for FQHE state at $\nu =\frac{1}{q}$ must contain the factor,

25 $$\begin{aligned} (z_i-z_{i+1})^q, \end{aligned}$$

for pairs of nearest-neighbouring electrons. This factor (25) must have this form as at exchanges of arguments of the multiparticle wave function, $z_i$ and $z_{i+1}$, it must contribute multiplicatively to this wave function in the unique way to produce the phase shift $e^{iq\pi }$. The braid from the cyclotron subgroup, which describes transposition of two arbitrary electrons *i*-th and *j*-th ones, must be of the following form,

26 $$\begin{aligned} \sigma _i^q\sigma _{i+1}^q\sigma _{i+2}^q\dots \sigma _{j-1}^q\left( \sigma _{j-2}^q\right) ^{-1}\dots \left( \sigma _{i+2}^q\right) ^{-1}\left( \sigma _{i+1}^q\right) ^{-1} \left( \sigma _{i}^q\right) ^{-1}, \end{aligned}$$

because here $\sigma _i^q$ are now generators instead of $\sigma _i$. This braid is visualized in Fig. 6b for $q=3$. We see that the scalar unitary representation of the braid (26) is $e^{iq\pi }$ for electrons, which forces a factor,

27 $$\begin{aligned} (z_i-z_{j})^q \end{aligned}$$

in the polynomial part of the function (13). In total this polynomial must be thus of the form,

28 $$\begin{aligned} \prod _{i,j;i>j}^N(z_i-z_j)^q, \end{aligned}$$

which completes the formal derivation of the Laughlin function.

## Derivation of multiparticle wave functions for FQHE states in the LLL for an arbitrary $\nu$ from FQHE hierarchy

For identical indistinguishable particles their numeration is arbitrary in principle. Without any loss of generality one can consider that $(i+1)$-th particle is a nearest neighbour (in the sense of the classical Wigner crystal at $T=0$ K) of *i*-th one. It is sufficient to note that it can hold for a selected *i* and thus for all *N* particles as for each particle in the Wigner lattice it exists its nearest neighbour. Because of indistinguishability the problem of conventional numeration of electrons on the plane is loosen of its significance. The enumeration of indistinguishable electrons is not intuitive. The problems with enumeration of conventional distinguishable particles disappear here. Similarly with next-nearest neighbours. For indistinguishable electrons important are only the integers $x_{\alpha }=\frac{N}{N_{\alpha }}$, where $N_{\alpha }$ is the number of next-nearest neighbours, which create the Wigner sublattice of $\alpha$-rank next-nearest neighbours. In 2D there exist only two types of classical Wigner lattice^3^—the hexagonal (of regular triangles) and regular (of squares), the latter energetically unstable. Thus sublattices must belong also to these classes. For hexagonal structure $x_{\alpha }=3,4,7,9,..$. Though the regular lattice is of higher energy than hexagonal one, its sublattice of next-nearest neighbour of first rank offers $x_{\alpha }=2$ not attainable in hexagonal lattice case. This needs some rearrangement of hexagonal lattice but it is convenient energetically as the first rank next-nearest neighbours in hexagonal stronger reduces the electron subset to $x_{\alpha }=3$. Finally, $x_{\alpha }=2,3,4,7,9,\ldots$, cf. Ref.^8^ for more detail.

The general homotopy invariant for cyclotron electron correlations of 2D electrons has the form^8^,

29 $$\begin{aligned} \frac{BS}{N}=\frac{hc}{x_1e}\pm \frac{hc}{x_2e}\pm \dots \pm \frac{hc}{x_qe}, \end{aligned}$$

where *q* is the number of loops of cyclotron orbit and $x_i$ indicates the index (one of numbers $x_{\alpha }$) of next-nearest neighbours in Wigner lattice commensurate with *i*-th loop. The form of the invariant (29) results from the commensurability condition of singleloop cyclotron orbit with next-nearest neighbours of rank $\alpha$ (the number of this next-neighbours is $N_{\alpha }=N/x_{\alpha }$), $\frac{BS}{N/x_{\alpha }}=\frac{hc}{e}$, or $\frac{BS}{N}=\frac{hc}{x_{\alpha }e}$. The extension of this onto *q*-loop orbit gives equation (29). Taking the general commensurability condition (29) for *q*-loop cyclotron orbit with nearest neighbours only, the invariant can be written as (neglecting ± in favour of $+$ only),

30 $$\begin{aligned} \frac{BS}{N}=\frac{qhc}{e}=\frac{hc}{e}+\dots +\frac{hc}{e}, \end{aligned}$$

where the latter sum has *q* components, which coincides with (24). The signs ± in (29) indicate a possible inverted (−) or congruent ($+$) circulation of a particular loop with respect to the preceding one.

To the invariant (29) it corresponds the filling fraction,

31 $$\begin{aligned} \nu =\left( \frac{1}{x_1}\pm \frac{1}{x_2}\pm \dots \pm \frac{1}{x_q}\right) ^{-1}, \end{aligned}$$

as $\nu =\frac{N}{N_0}$ and the degeneracy of LLs, $N_0=\frac{BSe}{hc}$. The filling fraction hierarchy of composite fermions, $\nu =\frac{y}{(q-1)y\pm 1}$, with $q=1,3,5,7$ and $y=1,2,3,\dots$^13^, is the specific case of (29) and is given by (31) for $x_1=\dots =x_{q-1} =1$, $x_q=y$ and ± before only last term.

To the invariant (29) there correspond generators of a particular cyclotron subgroup in the following form,

32 $$\begin{aligned} \begin{array}{l} b_j= (\sigma _j\sigma _{j+1}\dots \sigma _{j+x_{1}-2}\sigma _{j+x_1-1}\sigma _{j+x_1-2}^{-1}\dots \sigma _j^{-1})\\ ( \sigma _j\sigma _{j+1}\dots \sigma _{j+x_{2}-2}\sigma _{j+x_2-1}\sigma _{j+x_2-2}^{-1}\dots \sigma _j^{-1})^{\pm 1}\\ \dots \\ (\sigma _j\sigma _{j+1}\dots \sigma _{j+x_{q}-2}\sigma _{j+x_q-1}\sigma _{j+x_q-2}^{-1}\dots \sigma _j^{-1})^{\pm 1},\\ j=1,\dots , N',\;\;N'=N-max(x_i),\\ \end{array} \end{aligned}$$

where the segment,

33 $$\begin{aligned} (\sigma _j\sigma _{j+1}\dots \sigma _{j+x_{i}-2}\sigma _{j+x_i-1}\sigma _{j+x_i-2}^{-1}\dots \sigma _j^{-1}) \end{aligned}$$

corresponds to exchange of the electron *j*-th with $(j+x_i)$-th one along the *i*-th loop of *q*-loop cyclotron orbit and the fraction $\frac{1}{x_i}\le 1$ (as $x_i$ is one of integers $x_{\alpha }$) denotes here the fraction of next-nearest neighbours nested with this loop. For $x_i=1$ (the nearest neighbours) this whole segment (33) is simply $\sigma _j$.

The generators (32) define elementary exchanges of particles. Not all transpositions are possible but only those defined by the generators. Scalar unitary representations of generators (32) are $e^{i (1\pm 1 \pm \dots \pm 1 )\pi }$, as for original fermionic electrons we had chosen $\sigma _j\rightarrow e^{i\pi }$ and $\sigma _j^{-1}\rightarrow e^{-i\pi }$. The exponent in $e^{i (1\pm 1 \pm \dots \pm 1 )\pi }$ is always odd number multiplied by $\pi$ because *q* is odd.Therefore, the segment (33) must induce the factor to the multiparticle wave function,

34 $$\begin{aligned} \prod _{j=1,k=1;j<mod(j,x_i,1)+(k-1)x_i}^{N', N/x_i} (z_j-z_{mod(j,x_i,1)+(k-1)x_i}), \end{aligned}$$

($N'$ is the collection of admissible values of *j* at which the generator (32) can be defined, it is equal to $N-max(x_i)$ for $x_i$ entering (32)) as the projective scalar unitary representation of this segment is $e^{i\pi }$ (or $e^{-i \pi }$ if it enters as inverted operator). In the above formula $mod(j,x_i,1)$ is the rest of the division of *j* by $x_i$ with offset 1. Thus the total multiparticle wave function corresponding to generators (32) acquires the form,

35 $$\begin{aligned} \begin{aligned} \Psi (z_1, \dots , z_N)&= {\mathscr {A}} \prod _{j=1,k=1;j<mod(j,x_1,1)+(k-1)x_1}^{N', N/x_1} (z_j-z_{mod(j,x_1,1)+(k-1)x_1}) \\&\quad \times \, \prod _{j=1,k=1;j<mod(j,x_2,1)+(k-1)x_2}^{N', N/x_2} (z_j-z_{mod(j,x_2,1)+(k-1)x_2})\\&\quad \times \, \dots \\&\quad \times \, \prod _{j=1,k=1;j<mod(j,x_q,1)+(k-1)x_q}^{N', N/x_q} (z_j-z_{mod(j,x_q,1)+(k-1)x_q}) \\&\quad \times \, e^{-i\sum _{i=1}^N|z_{i}|^2/4l_B^2}, \end{aligned} \end{aligned}$$

for both two possibilities of scalar unitary representations related to ± in (29) causing only unimportant change of sign.

One can notice that in the case of $x_1=x_2=\dots =x_q=1$, the Laughlin function (11) is reproduced from (35). The envelope part of function (35), $e^{-i\sum _{i=1}^N|z_{i}|^2/4l_B^2}$, is correct only in GaAs (where the gaseous envelope is assumed) and this envelope changes in graphene according to explicit form of single electron LL functions in graphene (due to crystal field in graphene).

### Simple examples

**Figure 7:**
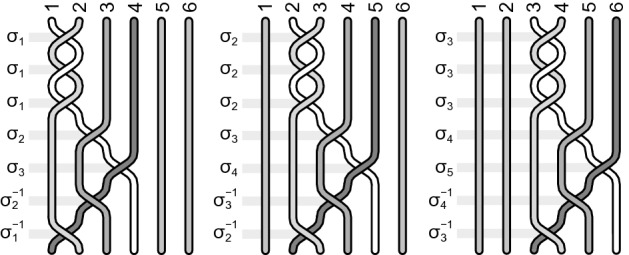
The generators for the homotopy pattern (1, 1, 3) at $\nu =\frac{3}{7}$ and $N=6$.

It is instructive to write out explicitly some elementary examples of the function (35). For $q=3$, $x_1=x_2=1$ and $x_3=2$ the filling fraction (31) is, $\nu =(1+1+1/3)^{-1}=3/7$, the generators are given by Eq. (32) and the wave function by Eq. (35). For $N=6$ this wave function has the explicit form,

36 $$\begin{aligned} \Psi _{3/7}(z_1,z_2,z_3,z_4)= & {} {\mathscr {A}} (z_1 - z_2)^2 (z_1 - z_3)^2 (z_2 - z_3)^2 (z_1 - z_4)^3 \nonumber \\&\quad \times \, (z_2 - z_4)^2 (z_3 - z_4)^2 (z_1 - z_5)^2 (z_2 - z_5)^3 (z_3 - z_5)^2 (z_1 - z_6)^2 \nonumber \\&\quad \times \, (z_2 - z_6)^2 (z_3 - z_6)^3 e^{-\sum _{i=1}^4|z_i|^2/4l_B^2}, \end{aligned}$$

which is apparently antisymmetric for admissible particle exchanges according to the generators (32), which in this case have the form,

37 $$\begin{aligned} b_j= \sigma _j^2 \sigma _j\sigma _{j+1}\sigma _{j+2}\sigma _{j+1}^{-1}\sigma _j^{-1}=\sigma _j^3\sigma _{j+1}\sigma _{j+2}\sigma _{j+1}^{-1}\sigma _j^{-1}, \end{aligned}$$

for $j=1,2,3$. These generators are illustrated in Fig. 7. No other electron transpositions are admitted in this case, i.e., for homotopy invariant given by Eq. (29) with $q=3$, $x_1=x_2=1$ and $x_3=3$ for $N=6$. Function (36) is not antisymmetric for arbitrary permutations of electron indices because they are not elements of the cyclotron braid subgroup for the homotopy pattern $\{1,1,3\}$—this subgroup (for $N=6$) is generated by only 3 generators (37), at action of which the function (36) transforms according their scalar unitary representation (assuming that original $\sigma _i\rightarrow e^{i\pi }$).

**Figure 8:**
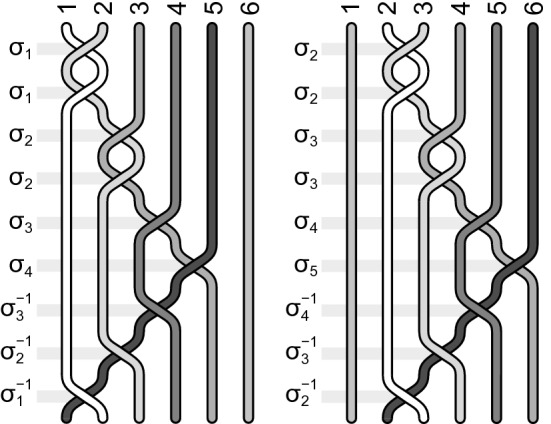
The generators for the homotopy pattern (1, 2, 4) at $\nu =\frac{4}{7}$ and $N=6$.

For another example let us take $\nu =\frac{4}{7}=(1+1/2+1/4)^{-1}$, i.e., $q=3$, $x_1=1$, $x_2=2$, $x_3=4$. From Eqs (35) and (32) we get for $N=6$,

38 $$\begin{aligned} \Psi _{4/7}(z_1,z_2,z_3,z_4,z_5,z_6)= & {} {\mathscr {A}} (z_1 - z_2) (z_1 - z_3)^2 (z_2 - z_3) (z_1 - z_4)\nonumber \\&\quad \times \,(z_2 - z_4)^2 (z_1 - z_5)^2 (z_2 - z_5) (z_1 - z_6) (z_2 - z_6)^2 e^{-\sum _{i=1}^6 |z_i|^2/4l_B^2} \end{aligned}$$

and related generators,

39 $$\begin{aligned} b_j=&\, \sigma _j\sigma _j\sigma _{j+1}\sigma _j^{-1}\sigma _j\sigma _{j+1}\sigma _{j+2}\sigma _{j+3}\sigma _{j+2}^{-1}\sigma _{j+1}^{-1}\sigma _j^{-1}\nonumber \\= &\,\sigma _j^2\sigma _{j+1}^2\sigma _{j+2}\sigma _{j+3}\sigma _{j+2}^{-1}\sigma _{j+1}^{-1}\sigma _j^{-1}, \end{aligned}$$

for $j=1,2$. Function (38) is apparently antisymmetric for admissible transpositions of electrons defined by generators (39). No other exchanges are possible at the filling fraction $\nu =\frac{4}{7}$ for the homotopy invariant (29) with $q=3$, $x_1=1$, $x_2 =2$ and $x_3=4$ at $N=6$ (cf. Fig. 8). Note that polynomials in (36) and (38) are homogeneous as required.

For multiparticle wave functions (35) one can assess the energy. In order to assess the energy corresponding to multiparticle trial wave function the contribution to energy of mutual electron interaction as well as of interaction with positive jellium must be accounted for. For the geometry of a disc with the radius $r=\frac{R}{l_B}=\sqrt{\frac{2N}{\nu }}$ in units of $l_B=\sqrt{\frac{\hbar c}{eB}}$ (where $\nu =\frac{N}{N_0}$, $N_0=\frac{BSe}{hc}$ and $\rho =\frac{\nu }{2\pi }$—the density of electrons $\frac{N}{S}$ when *S* is expressed in $l_B^2$ units), to the energy per single electron will contribute^36^,

- the energy of electrostatic interaction of the uniform jellium with itself,
  40 $$\begin{aligned} E_{jj}=\frac{\rho ^2}{N}\int _S d^2{{\mathbf {r}}}\int _S d^2{{\mathbf {r}}'}\frac{e^2}{|{\mathbf {r}}-{\mathbf {r}}'|}=\frac{8}{3\pi }\sqrt{\frac{\nu N}{2}}\frac{e^2}{l_B}, \end{aligned}$$
- the energy of interaction of the positive uniform jellium with electrons distributed according to the multiparticle wave function,
  41 $$\begin{aligned} E_{je}= & {} -\frac{1}{N}\left\langle\Psi ({\mathbf {r}}_1,\dots ,{\mathbf {r}}_N)\left|\rho \int _S d^2{\mathbf {r}}\sum _{i=1}^N\frac{e^2}{|{\mathbf {r}}-{\mathbf {r}}_i|}|\Psi ({\mathbf {r}}_1, \dots , {\mathbf {r}}_N)\right\rangle\right.\nonumber \\= & {} -\sqrt{2\nu N}\left\langle\Psi ({\mathbf {r}}_1,\dots ,{\mathbf {r}}_N)\left|\frac{1}{N}\sum _{i=1}^NF(r_i/r)|\Psi ({\mathbf {r}}_1, \dots , {\mathbf {r}}_N)\right\rangle\right. \frac{e^2}{ l_B}, \end{aligned}$$
- the energy of interaction of electrons with electrons,
  42 $$\begin{aligned} E_{ee}=\frac{1}{N}\left\langle\Psi ({\mathbf {r}}_1,\dots ,{\mathbf {r}}_N)\left| \sum _{i<j}^N\frac{e^2}{|\mathbf {r_i}-{\mathbf {r}}_j|}|\Psi ({\mathbf {r}}_1, \dots , {\mathbf {r}}_N)\right\rangle\right., \end{aligned}$$

where $$F(u)=\left\{ \begin{array}{ll} \frac{2E(u^2)}{\pi },&{}\quad for\; u<1,\\ _2F_1(\frac{1}{2},\frac{1}{2};2;\frac{1}{u^2}), &{}\quad for \;u\ge 1,\\ \end{array}\right.$$ here *E*(*x*) is the complete elliptic integral, and $_2F_1(a,b;c;x)$ is the hypergeometric function. $E_{jj}$, $E_{je}$, $E_{ee}$—the energies of jellium-jellium, jellium-electron and electron-electron interactions, respectively, are all calculated per single electron in the correlated state $\Psi ({\mathbf {r}}_1, \dots , {\mathbf {r}}_N)$. The energy $E_{jj}$ is taken analytically (is independent of electron distribution), whereas $E_{je}$ and $E_{ee}$ depend on the electron distribution defined by the appropriate multi-electron wave functions (for particular homotopy patterns, cf. Eq. (35)) and can be estimated by Metropolis Monte Carlo method of calculation of integrals with multi-argument integrand. The activation energy in the state $\Psi ({\mathbf {r}}_1,\dots ,{\mathbf {r}}_N)$ (per single particle and in units $\frac{e^2}{ l_B}$ at Gauss unit system) equals to $E=E_{jj}+E_{je}+E_{ee}$. For the exemplary homotopy phases presented above with the wave functions explicitly written at $N=6$ in Eqs (36) and (38), these energies calculated for larger *N* are listed in Tables 1 and 2.

**Table 1:** Activation energy for exemplary homotopy pattern $\{x_i\}=(1,1,3)$ for $\nu =\frac{3}{7}$ of FQHE correlations of composite fermion type (with $q=3$ and $x_1=x_2=1$ and $x_3=3$).

energy $\left[ \frac{e^2}{ l_B}\right]$	$N=15$	$N=20$	$N=30$	$N=40$	$N=50$
$E_{jj}$	1.52181	1.75724	2.15217	2.48511	2.77844
$E_{je}$	− 3.07717	− 3.55714	− 4.32402	− 4.99321	− 5.58363
$E_{ee}$	1.20496	1.42615	1.77256	2.09566	2.38900
*E*	− 0.350396	− 0.373753	− 0.399290	− 0.412444	− 0.416203

**Table 2:** Activation energy for exemplary homotopy pattern $\{x_i\}=(1,2,4)$ for $\nu =\frac{4}{7}$ of FQHE correlations not of composite fermin type (with $q=3$ and $x_1$, $x_2=2$ and $x_3=4$).

energy $\left[ \frac{e^2}{ l_B}\right]$	$N=15$	$N=20$	$N=30$	$N=40$	$N=50$
$E_{jj}$	1.75724	2.02908	2.48511	2.86956	3.20826
$E_{je}$	− 3.61860	− 4.10162	− 5.01700	− 5.76466	− 6.44849
$E_{ee}$	1.53389	1.70839	2.13082	2.47066	2.80887
*E*	− 0.327470	− 0.364154	− 0.401065	− 0.424442	− 0.431358

From Tables 1 and 2 we can notice that the activation energy grows with the increase of *N* in a similar manner as it has been demonstrated for the Laughlin functions^36^. Some other examples of various homotopy phases, their generators, wave functions and activation energies are presented in Ref.^22^.

## Conclusion

We have proved with the mathematical rigour that the derivation of the Laughlin function unavoidably must contain a topological element. This function cannot be derived within the local quantum mechanics (i.e., without nonlocal topological homotopy-type conditions imposed on Hamiltonian eigen-equation), which elucidates why the proof for the Laughlin ansatz was absent. Inclusion of multiloop cyclotron orbits with loops which can nest with next-nearest neighbours in the Wigner lattice shows how to generalize the Laughlin function onto more complicated cases protected by corresponding other homotopy invariants.

## Appendix 1: The role of scalar unitary representations of braid groups

The central role of scalar unitary representations of the braid group can be understood within the Feynman path integral quantization approach to multiparticle systems^37–39^. For *N* indistinguishable identical particles the Feynman path integral has the form^24,37^,

43 $$\begin{aligned} I({\mathbf {r}}_1,\dots , {\mathbf {r}}_N, t; {\mathbf {r}}'_1,\dots , {\mathbf {r}}'_N, t') =\sum _{l\in \pi _1(F_N)}e^{i\alpha _l}\int d\lambda _l e^{iS[\lambda _l({\mathbf {r}}_1,\dots , {\mathbf {r}}_N, t; {\mathbf {r}}'_1,\dots , {\mathbf {r}}'_N, t')]/\hbar }. \end{aligned}$$

$I({\mathbf {r}}_1,\dots , {\mathbf {r}}_N, t; {\mathbf {r}}'_1,\dots , {\mathbf {r}}'_N, t')$ is the propagator, which is defined as the matrix element of the evolution operator of the total *N*-particle system in the position representation. This propagator determines the probability amplitude (complex in general) for quantum transition in the multi-particle coordination space $F_N$, from point, ${\mathbf {r}}_1,\dots , {\mathbf {r}}_N$, in time instant *t* to the other point, ${\mathbf {r}}'_1,\dots , {\mathbf {r}}'_N$, in time instant $t'$. $d\lambda _l$ denotes the measure in the path space sector numerated by *l*-th element of the braid group $\pi _1(F_N)$ (*l* is a discrete index as braid groups are always countable). $S[\lambda _l({\mathbf {r}}_1,\dots , {\mathbf {r}}_N, t; {\mathbf {r}}'_1,\dots , {\mathbf {r}}'_N, t')]$ in the above formula is the classical action for the trajectory $\lambda _l$ joining selected points in the configuration space $F_N$ and lying in *l*-th sector of the trajectory space, i.e., with the *l*-th braid loop attached to an open trajectory joining start and final points in $F_N$. Such a structure of (43) results from the fact that to any open trajectory in $F_N$ can be attached (in arbitrary time instant between *t* and $t'$) an arbitrary closed loop form the braid group. As braids are nonhomotopic, thus the whole domain of the path integral decomposes into disjoint sectors numbered by braid group elements. Discontinuity between these sectors precludes the definition of an uniform measure in the whole domain of trajectories. Instead, the separate measures $d\lambda _l$ must be defined in disjoint nonhomotopic sectors of the path domain, and the summation over sectors accounts for their contributions to the total integral (43). However, each sector can contribute to the sum with the separate unitary (due to causality) scalar weight factor $e^{i\alpha _l}$. These weight factors establish a scalar unitary representation of the braid group^30^. Each distinct such a representation of the braid group defines thus a distinct quantization of the same classical multiparticle system. As the braids define exchanges of particles in $F_N$, thus their scalar unitary representations assign quantum statistics in the system.

As was proved by Sudarshan^26,28^ a specific scalar unitary representation of a particular braid defines a phase shift of the multiparticle wave function $\Psi ({\mathbf {r}}_1,\dots ,{\mathbf {r}}_N)$ when the arguments of this function, ${\mathbf {r}}_1, \dots , {\mathbf {r}}_N$ (i.e., classical positions of particles on the manifold *M*) exchange themselves mutually according to this braid.

## Appendix 2: Summary of the proof that braids $\sigma _i^q$ have larger size than $\sigma _i$

The Bohr–Sommerfeld rule links the area of the 1D phase space ranged by the closed classical phase trajectory with the corresponding number of quantum states. The quasiclassical wave function for a particle in an arbitrary 1D well *U*(*x*) can be written in equivalent forms starting from opposite turning points *a* and *b*^40^,

44 $$\begin{aligned} \Psi (x)=\left\{ \begin{array}{l} \frac{c}{\sqrt{p}} sin \frac{1}{\hbar }\int _a^xpdx,\;\; \text{for} \;\Psi (a)=0,\\ \frac{c'}{\sqrt{p}} sin \frac{1}{\hbar }\int _b^xpdx,\;\; \text{for} \;\Psi (b)=0,\\ \end{array}\right. \end{aligned}$$

where $p(x)=\sqrt{2m(E-U(x))}$ and for simplicity assuming vertical infinite borders of the well. From the uniqueness of the wave function requirement one gets,

45 $$\begin{aligned} 2 \int _a^bpdx= \oint p dx= S_{xp}= n 2\pi \hbar =n h. \end{aligned}$$

This is the Bohr–Sommerfeld quantization rule (*h* is the Planck constant, *n* is an integer; for arbitrary non infinite vertical borders, $S_{xp}=(n+\frac{1}{2})h$^40^). The formula (45) has been derived upon the condition that the trajectory between turning points is loopless. However, for a different homotopy class where only multiloop trajectories are available one obtains,

46 $$\begin{aligned} 2 \int _a^bpdx= \oint pdx= S_{px}= (2k+1) n 2\pi \hbar =n (2k+1) h, \end{aligned}$$

for a trajectory (*a*, *b*) with additional *k* loops in the simplest trajectory (i.e., when loopless trajectories are not available). It is easy to notice that each loop of all 2*k* loops pinned symmetrically (by *k* loops) to both branches, ’upper’ ($+p$) and ’lower’ ($-p$), of the full closed phase trajectory between *a* and *b* turning points in the integral $\oint pdx$ adds $2\pi$.

The Bohr–Sommerfeld rule can be applied to the effective 1D phase-space ($Y,P_y$) created by *x*, *y* components of the kinematic momentum of a 2D particle in the presence of a perpendicular magnetic field. The kinematic momentum components (at the Landau gauge choice, $\mathbf{A }=(0,Bx,0)$),

47 $$\begin{aligned} P_x= & {} -i\hbar \frac{\partial }{\partial x},\nonumber \\ P_y= & {} -i\hbar \frac{\partial }{\partial y} -\frac{e}{c}Bx, \end{aligned}$$

do not commute,

48 $$\begin{aligned} {[}P_x,P_y]_-= i\hbar \frac{e}{c}B. \end{aligned}$$

This commutator does not depend on the magnetic field gauge choice. Because of (48), the pair of operators, $Y=\frac{c}{eB}P_x$ and $P_y$, can be treated as operators of canonically conjugated generalized position *Y* and momentum $P_y$, for which $[Y,P_y]_- = i\hbar$. The 1D effective phase space, $(Y,P_y)$, is in fact the 2D space, $(P_x,P_y)$. Each closed trajectory in 2D kinematic momentum space is, on the other hand, the renormalized by the factor $\frac{c^2}{(eB)^2}$ and turned in plane by $\pi /2$, the corresponding trajectory in 2D space (*x*, *y*), which is clear by virtue of the quasiclassical Lorentz force formula, ${\mathbf {F}}=\frac{d{\mathbf {P}}}{dt}=\frac{e}{c}\frac{d{\mathbf {r}}}{dt}\times {\mathbf {B}}$, as it gives relation between both trajectory shifts, $dP_{x}=\frac{e}{c}B dy$ and $dP_y=-\frac{e}{c}Bdx$.

In 2D position space, trajectories (*x*, *y*) may belong to distinct homotopy classes and may be obligatory attributed to non-contractible additional loops (as happens in interacting multi-electron planar systems exposed to a sufficiently strong magnetic field, for which loopless braid trajectories are excluded as too short). Only possible trajectories are thus with additional loops. If multiloop trajectory is taken as the simplest trajectory between turning points, then the Bohr–Sommerfeld rule (with multiloop trajectories between turning points) gives,

49 $$\begin{aligned} S_{YP_y}= n (2k+1) hc, \end{aligned}$$

or rewritten to (*x*, *y*) space,

50 $$\begin{aligned} S_{x,y}=\frac{(2k+1)n hc}{eB}. \end{aligned}$$

The above formula defines the effective quantum of the magnetic field flux, for $\Delta n=1$,

51 $$\begin{aligned} \Phi _k=\Delta S_{x,y} B=\frac{(2k+1)hc}{e}, \end{aligned}$$

$\Delta S_{x,y}$ denote here the smallest change of $S_{x,y}$ in Eq. (50), when *n* is changed by 1. Only for $k=0$, i.e., for the homotopy class without additional loops in elementary braids (here taken as trajectories between turning points), the magnetic flux quantum equals to its fundamental lowest value $\Phi _0=\frac{hc}{e}$. For $k>0$, the homotopy constraints and resulted correlations topologically protected force the effective flux quantum to be $2k+1$ multiple of the fundamental one.

For $q=(2k+1)$-loop cyclotron orbits (or braids with *k* additional loops) the size of orbit is thus $\Delta S_{xy}= \frac{qhc}{eB}$, i.e., is *q* times larger than singleloop orbit $\frac{hc}{eB}$.

The quasiclassical method of the Bohr–Sommerfeld rule applied to many-particle systems is independent of particle interaction as the quasiclassical approach is not of perturbative type. Hence, the values of effective quanta of magnetic flux and related size of cyclotron orbits are also interaction independent.
